# Diagnostic Accuracy of Age and Alarm Symptoms for Upper GI Malignancy in Patients with Dyspepsia in a GI Clinic: A 7-Year Cross-Sectional Study

**DOI:** 10.1371/journal.pone.0039173

**Published:** 2012-06-13

**Authors:** Hooman Khademi, Amir-Reza Radmard, Fatemeh Malekzadeh, Farin Kamangar, Siavosh Nasseri-Moghaddam, Mattias Johansson, Graham Byrnes, Paul Brennan, Reza Malekzadeh

**Affiliations:** 1 Digestive Disease Research Institute, Shariati Hospital, Tehran University of Medical Sciences, Tehran, Iran; 2 International Agency for Research on Cancer, Lyon, France; 3 Department of Public Health Analysis, School of Community Health and Policy, Morgan State University, Baltimore, Maryland, United States of America; Howard University, United States of America

## Abstract

**Objectives:**

We investigated whether using demographic characteristics and alarm symptoms can accurately predict cancer in patients with dyspepsia in Iran, where upper GI cancers and *H. pylori* infection are common.

**Methods:**

All consecutive patients referred to a tertiary gastroenterology clinic in Tehran, Iran, from 2002 to 2009 were invited to participate in this study. Each patient completed a standard questionnaire and underwent upper gastrointestinal endoscopy. Alarm symptoms included in the questionnaire were weight loss, dysphagia, GI bleeding, and persistent vomiting. We used logistic regression models to estimate the diagnostic value of each variable in combination with other ones, and to develop a risk-prediction model.

**Results:**

A total of 2,847 patients with dyspepsia participated in this study, of whom 87 (3.1%) had upper GI malignancy. Patients reporting at least one of the alarm symptoms constituted 66.7% of cancer patients compared to 38.9% in patients without cancer (p<0.001). Esophageal or gastric cancers in patients with dyspepsia was associated with older age, being male, and symptoms of weight loss and vomiting. Each single predictor had low sensitivity and specificity. Using a combination of age, alarm symptoms, and smoking, we built a risk-prediction model that distinguished between high-risk and low-risk individuals with an area under the ROC curve of 0.85 and acceptable calibration.

**Conclusions:**

None of the predictors demonstrated high diagnostic accuracy. While our risk-prediction model had reasonable accuracy, some cancer cases would have remained undiagnosed. Therefore, where available, low cost endoscopy may be preferable for dyspeptic older patient or those with history of weight loss.

## Introduction

Dyspepsia, a condition defined as recurrent or persistent pain or discomfort centered in the upper abdomen, [1] affects 25%–40% of adults in the general population of the United States, incurring over $12 billion per year in direct annual costs in the United States and nearly £1 billion per year in the United Kingdom. [2]–[6] Several benign or malignant disorders may underlie dyspepsia, including esophagitis, gastroesophageal reflux disease (GERD), peptic ulcer disease (PUD), erosive duodenitis, [7] and most importantly upper gastrointestinal (UGI) malignancies, which are estimated to be responsible for 1%–3% of all cases of dyspepsia. [7]–[10] However, in over half of the dyspeptic patients no obvious structural abnormality can be found, a condition called “functional” or “non-ulcer” dyspepsia. [1], [11]–[13] Recently some experts have argued that GERD should be excluded from the etiologies of dyspepsia and treated as a different entity, [2], [14] but this is still in dispute. [15], [16]

There are several alternative strategies for initial management of dyspepsia including empirical acid suppressive therapy, *H. pylori* test and treat, and prompt endoscopy, [17], [18] and several studies have tried to find the best strategy. [11]–[13], [18]–[20] It has been suggested that the most cost-effective initial approach in primary care, particularly in countries with low rates of *H. pylori* infection is test and treat strategy. [17], [21]–[23] However, it may delay early diagnosis of malignant underlying disease beyond the point where it is still curable and also might not be practical in countries with very high rates of *H. pylori* infection, such as Iran. In addition, endoscopy is an accurate but costly method of early diagnosis of UGI malignancies, which are considered as the most important causes of global cancer deaths. [24] It may be cost-effective to stratify dyspeptic patients as high-risk and low-risk, and then perform immediate endoscopy on the high-risk group while applying other alternatives for the low-risk group. Thus some experts have recommended prompt endoscopy in newly diagnosed dyspeptic patients having any alarm symptoms including unintentional weight loss (>10% of body weight), dysphagia, GI bleeding, persistent vomiting, abdominal palpable mass and anemia, as well as in patients who are over age 50. [12], [19], [25]–[27] In contrast, several studies have shown limited predictive value for either alarm features or age to be able to differentiate low- and high-risk dyspeptic patients for underlying malignancies. [28]–[33] Prompt endoscopy in patients over 50 years regardless of alarm symptom status has been shown to increase the proportion of curable cases of UGI malignancies by as much as 30%, [34]–[36], but the cost-effectiveness of initial endoscopy in this age group for improving survival of cancer patients is uncertain. [36], [37] Distinct UGI malignancy incidence rates and various distributions of its topographical types in different populations [7]–[10] as well as differences in *H. pylori* infection rates [38], [39] could partly explain the variable results.

Gastric cancer, followed by esophageal cancer, is reported as the most common cancer in Iranian men. As well, *H. pylori* infection is highly prevalent (>80%) in the Iranian adult population. [39]–[45] Although acid peptic disease is also still common in Iran, [44], [46] the major indication for UGI endoscopy in Iran is ruling out upper GI malignancy as underlying cause. We have conducted a relatively large-scale study to assess the role of alarm symptoms and their diagnostic accuracy in predicting UGI malignancy in patients with dyspepsia in a country with high prevalence of *H. pylori* infection and upper GI malignancy. Through developing a risk-prediction model, we also tried to find a way to maximally use all information from age and alarm symptoms, altogether, to find high-risk individuals for UGI malignancy. To the best of our knowledge, no previous study investigated alarm symptoms in Western Asia and Middle East region.

## Methods

### Study population

All consecutive patients referred to Behrooz Clinic, a tertiary referral gastroenterology clinic in Tehran, and diagnosed with dyspepsia from 2002 to 2009 were invited to participate in this study. Patients with UGI malignancy previously diagnosed through other imaging tools, such as CT scan or barium swallow, and patients who had already a diagnosis of UGI cancer or undergone gastrectomy or esophagectomy (4 cases) were not included in this study. All study participants signed a written informed consent and Institutional Review Board of Digestive Disease Research Institute of Tehran University of Medical Sciences (TUMS) approved the study design and methods.

### Exposure assessment

Demographic and anthropometric characteristics, history of any alarm symptoms, family history of UGI malignancies in first degree relatives, and also data on cigarette smoking status were collected by interviewing the patients. Body mass (kg) and height (cm) were measured; body mass index (BMI) was calculated and categorized based on WHO recommendations. Pack-years (pys) of cigarette smoking were calculated by multiplying duration of smoking (in years) and daily use amount (in cigarettes per day divided by 20). Accordingly, we categorized all patients into four smoking groups: never smokers, ex-smokers (quit smoking more than a year before interview), current light smokers (less than 20 pys) and current heavy smokers (20 pys or more). Rapid urease test (RUT) was performed on all patients during endoscopy to detect *H. pylori* infection. Alarm symptoms in this study were unintentional weight loss (≥10% of body weight in recent 6 months), dysphagia (perception of an impediment to the normal passage of swallowed material), GI bleeding (any evidence of hematemesis and/or melena), and persistent vomiting (at least 7 to 10 days of protracted vomiting). [47], [48]

### Outcome measurement

All patients underwent prompt endoscopy using Olympus video-endoscopes (GIF type-160), while they were asked not to use proton pump inhibitors (PPIs) or H_2_ blockers for at least 2 weeks prior to endoscopy to avoid their masking effect on visibility of malignancy during endoscopy. [49] In case of any suspected malignancy, multiple biopsy specimens were taken from the suspected lesion and were sent to two separate pathology centers. All cancer diagnoses were histologically confirmed. UGI malignancy was defined as any histologically confirmed esophageal, gastric or duodenal cancer detected during endoscopy.

### Statistical Analysis

Using histology as the gold standard for diagnosis of UGI malignancies, we calculated sensitivity, specificity, positive predictive value (PPV), and negative predictive value (NPV). We also calculated and present functions of sensitivity and specificity, including positive diagnostic likelihood ratio (PDLR), negative diagnostic likelihood ratio (NDLR), and diagnostic odds ratio (OR), and their related 95% confidence intervals, as measures of diagnostic accuracy for each individual alarm symptom. [50]

We estimated odds ratios (ORs) and 95% confidence intervals for age, demographics, and each of alarm symptoms using univariable and multivariable adjusted logistic regression models. Adjusted model included age, gender, level of education, cigarette smoking, history of weight loss, GI bleeding, persistent vomiting and dysphagia. Based on regression model findings, we decided to report diagnostic accuracy measures for each alarm symptom, in four age categories: less than 36 years of age, between 36 and 49, between 50 and 65 and finally 65 or older, as the patients in each group were similar within the group and different from the other groups in terms of OR.

We developed a risk-prediction model, through a backward stepwise selection in a multivariable logistic regression analysis. We used all variables that showed significant association with UGI malignancy in univariable regression analysis, except for BMI and *H. pylori*. BMI was not included because of its collinearity with weight loss. *H. pylori* was not included because it was measured during endoscopy (and not prior to that), so it was not helpful to assess the need for endoscopy. We also included all possible two-way interaction terms between four alarm symptoms in the initial model. Subsequently, using an automated backward-stepwise multivariable method, we removed the predictors with highest p values on the basis of Wald test, so that achieving the final model that only comprised by the predictors with a multivariable p value of less than 0.05. Although forward selection gives a more parsimonious model, backward selection is generally preferable if stepwise selection is applied. [51] Since there were 46 missing values in two variables; family history of UGI cancer (17 patients) and level of education (29 patients) we performed the analyses on 2,801 remaining patients; 82 UGI malignancies. The *complete case analysis approach* was applied, because of the small percentage of missing values in only two variables that was considered to be completely at random.

As the rule for prediction model, we used the linear predictor, which is the sum of the products of regression coefficients with the corresponding variable values from the final logistic model, and for convenience added a constant value of 1.5 to ensure it was positive; *Risk Score = 1.5+(Regression Coefficient×Variable Value)*

**Figure 1:**
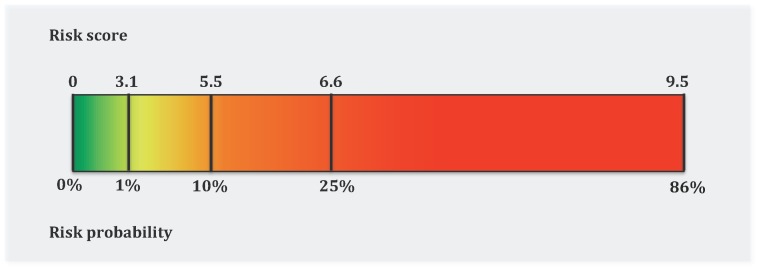
The risk scores and their corresponding risk probabilities derived from the suggested risk-prediction model.

The corresponding risk probability was calculated using the equation; *P = 1/(1+e^−ß^)*, where *ß = Constant+(Regression Coefficient×Variable Value)*; for example, probability (*P*) thresholds of 1%, 10% and 25% correspond to risk score (RS) cutoff levels of 3.1, 5.5 and 6.6, respectively (Figure 1).

Three percent of all patients had UGI malignancies, hence we chose thresholds of 1% and 10%, round numbers that were approximately three times lower and higher than the overall population average, to denote low- or high-risk, respectively. Subsequently, we defined four risk groups for UGI malignancy: low-risk group with a probability of less than 1%, intermediate-risk group with a probability between 1% to 10%, high-risk group with a probability of 10% to 25%, and excessive-risk group with a probability of higher than 25%.

**Figure 2:**
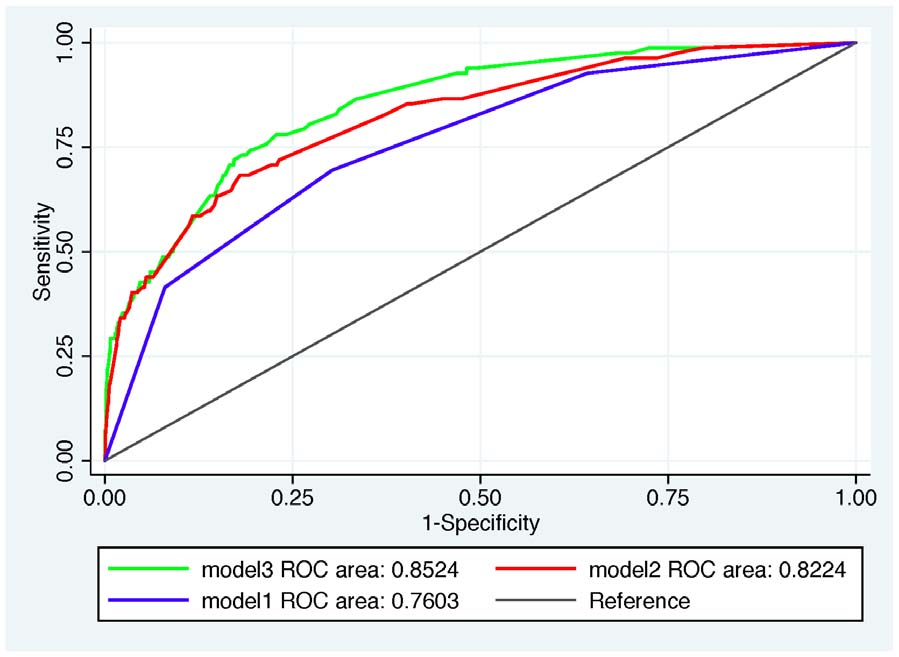
ROC curves based on three predictive model for upper GI malignancy in dyspeptic patients, including model1; only age included, model2; age plus alarm symptoms, and model3; the risk-prediction model.

Once the variables to be included in the model were defined, we examined the calibration of the model by performing Hosmer-Lemeshow goodness-of-fit test. We also assessed the overall performance of the model using Nagelkerke's *R* ^2^-as a measure of explained variation in log-likelihood scale, and Brier score (or average prediction error). [52] Finally, the discrimination capability of the model was estimated using the area under the *receiver operating characteristic* (ROC) curve or AUC and its 95% confidence intervals. To further evaluating the discrimination ability of our risk-prediction model, we compared it with two other models: 1) age only; 2) age plus alarm symptoms; in terms of AUC (Figure 2). We also plotted reclassification table and calculated net reclassification index (NRI) and integrated discrimination index (IDI), investigating added discriminatory performance of our suggested risk-prediction model compared to model 2. [53]–[58] In evaluating model performance, over-fitting is a well-known statistical phenomenon where a model will always perform better on the data used to construct it than when predicting from independent but similar data. While we had no data to externally validate our model, we used repeated 10% cross-validation to guard against over-fitting. [51], [52] In this procedure the model was fitted to a randomly selected 90% of the data then tested on the remaining 10%; the procedure then being repeated 10 times and the resulting statistics averaged.

All statistical analyses were conducted using STATA statistical software, version 11 (STATA Corp, College Station, TX) and all reported p-values are 2-sided.

## Results

A total of 2,847 patients with dyspepsia who were referred to Behrooz clinic, a tertiary GI clinic in Tehran, participated in this study. Table 1 shows the demographic characteristics, as well as habits and distribution of alarm symptoms in study participants. The mean (±SD) age of the participants was 42.5 (±15.0), and approximately half (50.5%) were females. Of these, 1,131 patients (39.7%) had at least one alarm symptom; the most common reported alarm symptom was dysphagia (n = 547; 19.2%).

UGI malignancies were histologically confirmed in 87 (3.1%) cases. Compared to all patients with dyspepsia, patients with UGI malignancies were significantly older (mean age of 58.0 years); more likely to be male (60.9%); to have an education of less than high school diploma (52.4%); to have a family history of UGI malignancies (14.9%); to have ever smoked cigarettes (35.6%); and more likely to be positive for *H. pylori* (70.6%) (Table 1). Patients reporting at least one of the alarm symptoms constituted 66.7% of patients with UGI malignancies compared to 38.9% in patients without cancer (p value<0.001).

**Table 1:** Demographic characteristics, habits and distribution of alarm symptoms in all dyspeptic patients and who with upper GI malignancy.

	All patients with dyspepsia		Dyspeptic patients with UGI malignancy		*P value* *
	Frequency€	percentage	Frequency€	percentage	
Age (±SD)	42.5±15.0		58.0±15.4		<0.001
Gender					
Female	1,439	50.5	34	39.1	0.030
Male	1,408	49.5	53	60.9	
Education level					
Less than high school	743	26.4	43	52.4	<0.001
Higher education	2,075	73.6	27	47.6	
Body Mass Index (kg/m^2^)					
Underweight (<18.5)	146	5.4	2	2.6	0.008
Normal (18.5–24.9)	1,310	48.5	52	67.5	
Overweight (25–29.9)	965	35.7	19	24.7	
Obese (≥30)	282	10.4	4	5.2	
History of weight loss					
Negative	2,533	89.0	49	56.3	<0.001
Positive	314	11.0	38	43.7	
History of GI bleeding					
Negative	2,435	85.5	66	75.9	0.009
Positive	412	14.5	21	24.1	
History of persistent vomiting					
Negative	2,525	88.7	63	72.4	<0.001
Positive	322	11.3	24	27.6	
History of dysphagia					
Negative	2,300	80.8	61	70.1	0.010
Positive	547	19.2	26	29.9	
Family history of upper GI cancer					
Negative	2,583	91.3	74	85.1	0.037
Positive	247	8.7	13	14.9	
Cigarette smoking					
Never smoker	2,326	81.7	56	64.4	<0.001
Ex-smoker	102	3.6	5	5.8	
Current light smoker	328	11.5	13	14.9	
Current heavy smoker	91	3.2	13	14.9	
*H. pylori* test result‡					
Negative	1,492	53.1	25	29.4	<0.001
Positive	1,318	46.9	60	70.6	

€ Since we had some missing information in educational level, weigh, height, and *H. pylori* test results, the sum of frequencies in these variables is not equal to study sample size.

‡ *H. pylori* infection was detected based on Rapid Urease Test (RUT), during endoscopy.

* *P* values are calculated using independent sample t-test for age and chi-square tests for other variables to compare the distribution of the variables between patients with and without UGI malignancy.

Table 2 shows the endoscopic and histological findings in the study participants. The most common endoscopic findings were GERD (72.6%), followed by PUD (15.0%), and UGI malignancies (3.1%). The Los Angeles (LA) classification was used for the endoscopic diagnosis of GERD, which classifies it into 4 subgroups; A to D, according to number and length of observed mucosal breaks and involvement of one or more mucosal folds. [59] Of the 87 patients with cancer, 68 (78.2%) were diagnosed with gastric cancer, 16 (18.4%) with esophageal cancer, and 3 (3.4%) with duodenal cancer. The majority of all malignancies (54.0%) were well-differentiated. Esophageal cancers were located more in the middle-third of the esophagus (68.7%) and were more of squamous cell type (62.5%). The majority of gastric cancers were adenocarcinomas (88.2%) and were located in the antrum (35.3%). Of the 3 duodenal cancers, 2 (66.7%) were seen in D1. (Table 2)

**Table 2:** Endoscopic and histologic findings in study population.

		Frequency	Percent
GE Reflux Disease (GERD)			
No GERD		781	27.4
GERD-A		1,555	54.6
GERD-B		443	15.6
GERD-C		64	2.3
GERD-D		4	0.1
Peptic Ulcer Disease (PUD)			
No PUD		2,420	85.0
Duodenal ulcer		393	13.8
Gastric ulcer		27	0.9
Synchronous ulcer		7	0.3
Upper GI Malignancy			
No malignancy		2,760	96.9
Esophageal		16	0.6
Gastric		68	2.4
Duodenal		3	0.1
Cancer Grade			
Esophageal	Well-diff	12	75.0
	Intermediate-diff	4	25.0
	Poor-diff	0	0.0
Gastric	Well-diff	35	51.5
	Intermediate-diff	27	39.7
	Poor-diff	6	8.8
Duodenal	Well-diff	0	0.0
	Intermediate-diff	3	100
	Poor-diff	0	0.0
Cancer Morphology			
Esophageal	SCC	10	62.5
	Adenocarcinoma	6	37.5
Gastric	Adenocarcinoma	60	88.2
	MALT	8	11.8
Duodenal	Adenocarcinoma	3	100
Cancer Topography			
Esophageal	Upper third	2	12.5
	Middle third	11	68.7
	Lower third	3	18.8
Gastric	GE junction	4	5.9
	Cardia	14	20.6
	Corpus	22	32.3
	Antrum	24	35.3
	Diffuse	4	5.9
Duodenal	D1	2	66.7
	D2	1	33.3

Table S1 shows diagnostic values for alarm symptoms for all participants and by age category. The prevalence of UGI cancers by age category, from youngest to oldest, was 0.71%, 1.89%, 3.65%, and 14.3%, respectively. Due to increasing prevalence of cancer with age, PPV increased as a function of age. For example PPV for dysphagia was nearly 16-fold higher in the oldest versus the youngest group. Among alarm symptoms, weight loss was the strongest predictor of UGI cancers.

We calculated diagnostic values for experiencing only one, two, and more than two alarm symptoms, and at least one symptom, compared to patients who had never experienced any alarm symptoms, as reference group (Table S2). Positive predictive value increased with increasing number of reported alarm symptoms and older age, such that PPV increased from 0.70% in patients younger than 35 years of age with only one alarm symptom to 58.3% in patients older than 65 years of age with more than two alarm symptoms. Having at least one alarm symptom had the highest sensitivity (66.7%) but the lowest specificity (61.1%).

We used multivariable adjusted logistic regression models to study the independent diagnostic ORs for each alarm symptom (Table 3). In these models, age showed very high ORs, in both unadjusted and adjusted models, with OR (95%CI) of 22.8 (8.86–58.5), for the oldest (≥65 years old) compared to the youngest age group; a near 2-fold increase in cancer odds was observed for each 10 years increase in age. Men did not have significantly higher odds of UGI cancers than women. Heavy smokers were in a significantly higher risk of developing UGI malignancies compared to never-smokers (OR (95%CI): 5.07 (2.33–11.0)). Among alarm symptoms, weight loss was the leading predictive factor for UGI malignancy in adjusted models (OR (95%CI) = 4.89 (2.91–8.23)), while persistent vomiting with OR (95%CI) of 2.26 (1.27–4.03) was the second most important alarm symptom. Patients with UGI cancer were approximately twice as likely to have positive RUT results compared to patients without malignancy, mainly due to the association between *H. pylori* and gastric cancer (OR (95%CI) = 3.05 (1.65–5.64)).

As fully explained in the methods part, we developed a risk-prediction model to predict UGI malignancies in dyspeptic patients (Table 4). Figure 1 shows the risk score (RS) thresholds and their corresponding risk probabilities, as described in the methods section; low-risk group with a RS<3.1; intermediate-risk group with a 3.1≤RS<5.5; high-risk group with a 5.5≤RS<6.5; and excessive-risk group with a RS of ≥6.6. To provide some examples, a 35-year-old never-smoker who did not have any of the alarm symptoms had a risk score of 1.5 and was categorized as low-risk (<1% chance of UGI cancer), whereas a 65-year-old current heavy smoker with weight loss but no other alarm symptoms had a score of 7.9 ( = 1.5+3.3+1.3+1.8) and was therefore categorized as excessive-risk (>25% chance of cancer). Using RS = 2.2(or 5.5, or 6.6) as the cutoff levels, the estimated sensitivity and specificity was equal to 100% (or 42.0%, or 29.6%) and 24.5% (or 95.0%, or 98.9%), respectively (Table 5).

**Table 3:** Estimated odds ratios of demographic characteristics and alarm symptoms for upper GI malignancies, based on unadjusted and multivariable adjusted regression models.

	OR for Upper GI Cancers	
	Unadjusted model	Multivariable adjusted model€
Age categories		
≤35 years old	ref	ref
36–49 years old	2.83 (1.19–6.77)	4.13 (1.60–10.6)
50–64 years old	5.51 (2.36–12.9)	6.83 (2.68–17.4)
≥65 years old	23.4 (10.3–53.2)	22.8 (8.86–58.5)
Gender		
Female	ref	ref
Male	1.62 (1.04–2.50)	1.39 (0.82–2.38)
Education level		
Higher education	ref	ref
Less than high school	3.21 (2.06–4.99)	1.51 (0.89–2.57)
History of weight loss		
Negative	ref	ref
Positive	6.98 (4.49–10.8)	4.89 (2.91–8.23)
History of GI bleeding		
Negative	ref	ref
Positive	1.93 (1.17–3.19)	1.77 (1.01–3.10)
History of persistent vomiting		
Negative	ref	ref
Positive	3.15 (1.94–5.11)	2.26 (1.27–4.03)
History of dysphagia		
Negative	ref	ref
Positive	1.83 (1.15–2.93)	1.16 (0.66–2.05)
Family history of upper GI cancer		
Negative	ref	ref
Positive	1.88 (1.03–3.45)	2.00 (1.01–3.95)
Cigarette smoking		
Never smoker	ref	ref
Ex-smoker	2.09 (0.82–5.33)	0.88 (0.25–3.08)
Current light smoker	1.67 (0.90–3.09)	2.03 (1.02–4.04)
Current heavy smoker	6.75 (3.55–12.9)	5.07 (2.33–11.0)
Body mass index (Kg/m^2^)		
Under weight (<18.5)	0.34 (0.08–1.39)	0.48 (0.10–2.17)
Normal weight (18.5–24.9)	ref	ref
Over weight (25–29.9)	0.48 (0.28–0.83)	0.65 (0.36–1.18)
Obese (≥30)	0.35 (0.12–0.97)	0.59 (0.20–1.71)
*H. Pylori* test result‡		
Negative	ref	ref
Positive	2.80 (1.74–4.49)	2.08 (1.24–3.47)

€ Multivariable model is adjusted for age, gender, educational level, cigarette smoking and history of weight loss, GI bleeding, persistent vomiting and dysphagia.

‡ *H. pylori* infection is detected based on Rapid Urease Test (RUT), during endoscopy.

**Table 4:** Risk-prediction model for predicting risk of upper GI malignancy in dyspeptic patients.

	Regression Coefficient (95%CI)	*P* value
Age categories		
36–49 yrs old	1.3 (0.4 to 2.3)	0.006
50–64 yrs old	1.9 (1.0 to 2.9)	<0.001
≥65 yrs old	3.3 (2.4 to 4.2)	<0.001
Weight loss	1.3 (0.6 to 1.9)	<0.001
Persistent vomiting	0.9 (0.3 to 1.5)	0.003
GI bleeding	1.0 (0.4 to 1.6)	0.002
Weight loss×Dysphagia	1.1 (0.3 to 2.0)	0.012
GI bleeding×Dysphagia	−1.5 (−2.8 to −0.1)	0.037
Family history of upper GI cancer	0.7 (0.0 to 1.4)	0.048
Cigarette smoking		
Current light smokers	0.8 (0.1 to 1.5)	0.017
Current heavy smokers	1.8 (1.0 to 2.5)	<0.001
Constant	−6.2 (−7.1 to −5.3)	<0.001

**Table 5:** The diagnostic characteristics of choosing different risk score cut-off levels, derived from the risk-prediction model.

Thresholds for risk score	Sensitivity%	Specificity%	Correctly classified%	No. patients£	No. missing cancers€	Probability%¥
RS≥2.2	100	24.5	26.7	749	0	0.4
RS≥3.1	93.8	51.6	52.8	1,408	5	1
RS≥4.2	76.5	78.9	78.9	2,211	20	3
RS≥5.5	42.0	95.0	93.5	2,634	47	10
RS≥6.6	29.6	98.9	96.9	2,749	57	25

£ The number of patients that have a RS of less than the chosen threshold.

€ The number of cancer patients that have a RS of less than the chosen threshold and consequently are missed, due to not being selected for prompt endoscopy.

¥ The probability of having cancer when the RS is exactly equal to the threshold, according to mentioned formulae in the method section.

The suggested risk-prediction model, including 8 predictors, had the number of events per variable (EPV) of about 10, which indicates an acceptable sample size to provide an adequate risk-prediction model. [52] As shown in Table 6, a nonsignificant Hosmer-lemeshow test (p = 0.71) showed that this model adequately predicts, for each level of risk, the percentage of patients with the outcome (good calibration). The comparison of Brier score and Nagelkerke's *R* ^2^ measures in three models, suggested slightly better overall performance for the risk-prediction model. The Akaike information criterion (AIC) was in favor of the proposed risk-prediction model (model3); however, the Bayesian information criterion (BIC) didn't show any superiority for model3 versus model2. The net reclassification index (NRI) of 23% compared to model2, again advocated for the third model; however, the integrated discrimination index (IDI), which was calculated by subtracting discrimination slopes of compared models, indicated a minor improvement in discrimination ability of model3 versus model2; 4.3% (Table 6). The estimated AUC comparison showed a statistically significant higher discriminatory capacity of model3, though not substantially; the AUC (95% CI) of 0.852 (0.812–0.893) for risk-prediction model was significantly higher than both model2 (p = 0.022) with AUC (95% CI) of 0.822 (0.774–0.870) and model1 (p<0.001) with AUC (95% CI) of 0.760 (0.709–0.812) (Figure 2).

**Table 6:** Performance of three different models for predicting UGI malignancy in dyspeptic patients.

Performance measure	Prediction Models*		
	Model1	Model2	Model3
Overall			
Brier	0.027	0.025	0.024
Brier_scaled_ **	4.2%	12.0%	16.5%
*R* ^2^ (Nagelkerke)	12.9%	22.3%	26.9%
Discrimination			
Area under ROC	0.760 (0.709 to 0.812)	0.822 (0.774 to 0.870)	0.852(0.812 to 0.893)
Discrimination slope	0.043	0.115	0.158
Calibration			
Hosmer-Lemeshow test	*X* ^2^ (df:2) = 0.30	*X* ^2^ (df:6) = 2.90	*X* ^2^ (df:8) = 5.45
	P = 0.86	P = 0.82	P = 0.71
Global model fit			
Akaike information criterion (AIC)	663.5	607.4	583.9
Bayesian information criterion (BIC)	687.2	655.0	655.2
Reclassification			
Net improvement index (NRI)			
Model2 vs. Model1	22.3%		
Model3 vs. Model1	42.2%		
Model3 vs. Model2		23.3%	
Integrated discrimination index (IDI)			
Model2 vs. Model1	7.2%		
Model3 vs. Model1	11.5%		
Model3 vs. Model2		4.3%	

* Model1: age only; Model2: age plus four alarm symptoms (weigh loss, persistent vomiting, GI bleeding, dysphagia); Model3: risk-prediction model.

** Brier*_scaled_* = 1−Brier/Brier*_max_*, where Brier*_max_* = mean(p)*×*(1−mean(p)); and mean(p) is mean probability of outcome prediction based on model.

Using the marginal numbers of a reclassification table (Table 7), we evaluated the calibration of the risk-prediction model compared to model2, which demonstrated comparable predicted probabilities with observed proportions, except in the third group, with risk probabilities between 10% and 25%.

**Table 7:** Reclassification table for age and alarm symptom model (Model2) and risk-prediction model (Model3).

	Model2*	Model3*				
		<1%	1–9%	10–24%	> = 25%	Total
Event						
	<1%	2	1	0	0	3
	1–9%	3	40	1	2	46
	10–24%	0	1	8	4	13
	> = 25%	0	0	2	18	20
	**Total**	5	42	11	24	82
Nonevent						
	<1%	789	21	0	0	810
	1–9%	603	1,150	41	1	1,795
	10–24%	0	19	52	9	80
	> = 25%	0	2	15	17	34
	**Total**	1,392	1,192	108	27	2,719
All						
	<1%	791	22	0	0	813 (29.0)%event = 0.3
	1–9%	606	1,190	42	3	1841 (65.7)%event = 2.5
	10–24%	0	20	60	13	93 (3.4)%event = 13.4
	> = 25%	0	2	17	35	54 (1.9)%event = 37.0
	**Total (%)**	1,397 (49.9)%event = 0.3	1,234 (44.1)%event = 3.4	119 (4.2)%event = 9.2	51 (1.8)%event = 47.1	2801

* Model2: age plus four alarm symptoms (weigh loss, persistent vomiting, GI bleeding, dysphagia); Model3: risk-prediction model.

The results of repeated 10% cross-validation indicated that the estimated average of AUC from risk-prediction model (0.820; 95%CI: 0.764–0.876) was not largely different from the average AUC in validation set (0.796; 95%CI: 0.744–0.848); 0.024.

## Discussion

We studied age and several alarm symptoms to learn whether they can provide useful diagnostic information to classify dyspeptic patients, referred to a tertiary GI clinic, as high-risk and low-risk for UGI cancers.

In the adjusted models, older age, history of weight loss, history of GI bleeding, persistent vomiting, being current cigarette smoker, family history of UGI cancer, and *H. pylori* positivity were all positively associated with risk of UGI cancers. Of these, age and weight loss were the most important predictors. Other predictors, such as male sex, lower education, and history of dysphagia were also associated with higher risk in unadjusted models, but lost statistical significance in the adjusted models. Since we measured *H. pylori* infection by rapid-urease-test (RUT) during endoscopy, this variable wasn't included in risk-prediction model. Moreover, the majority of *H. Pylori* infected gastric cancer patients develop severe gastric atrophy before gastric cancer, making stomach environment unfavorable for *H. pylori* survival, and thus would become *H. pylori* negative by RUT.

Some previous studies have also assessed the value of age and alarm symptoms in predicting risk of cancer in dyspeptic patients. [28], [60], [61] Bai and colleagues studied the predictive value of alarm symptoms and age for UGI malignancy in China and found limited value for either age or any alarm symptoms. [61] In their study, alarm symptoms were highly specific but had low sensitivity. However, they based most of their discussion on PDLR of each symptom and did not build models using all predictor variables to predict risk of UGI malignancies. Performing a meta-analysis, Fransen and colleagues found limited diagnostic values including sensitivity, specificity and predictive values, for each individual alarm symptom, i.e., dysphagia, weight loss, bleeding, and vomiting. [33] They suggested using alarm symptoms in combination with other factors – such as age, gender, or smoking – might be a better tool for selection of high-risk patients; however they were unable to test their hypothesis. Kapoor *et al.*, [28] built a model using a number of alarm symptoms and age, and validated their model in another group of patients. Using a combination of symptoms, they were able to generate a model with high sensitivity and high NPV, but low specificity and low PPV, to predict risk of UGI malignancies. However, they did not use the weight of the symptoms based on their odds ratio and perhaps did not make use of the full extent of information in their dataset. Finally, Numans and colleagues [60] developed a risk-prediction model using calculated total scores and showed that classical alarm symptoms, via a risk-prediction model, are useful predictors of UGI malignancy. However, their model is somewhat complex and, with inclusion of several variables, somewhat unstable. Like the results of our study, nearly all of these studies showed relatively low value for each alarm symptom, but perhaps a number of unnecessary endoscopies could be avoided using a combination of symptoms.

Although we found several variables that were each associated with higher risk of having cancer, our results showed that no single predictor could perfectly differentiate between high- and low-risk groups; sensitivities and specificities for each of the predictors were far from one. In principle, simply adding the number of risk factors is not the most efficient use of data, as different risk factors predicted cancer with substantially different odds ratios. Therefore, the most appropriate way of predicting risk would be using the risk-prediction model. However, our results show that the proposed risk-prediction model was unable to provide any important improvement in prediction compare to a model based on including only age and the generally accepted alarm symptoms.

Our proposed risk-prediction model was not perfect, despite acceptable overall model fit and calibration. However, such model could somewhat adequately discriminate patients in our setting into a wide range, with risks less than 1% to risks over 25%, with acceptable calibration. Given that all of the predictors used in this risk-prediction model could easily be obtained from a simple questionnaire, this might provide useful information for the physician in deciding whether to perform immediate endoscopy or to first try empiric forms of treatment.

Some predictors of cancer, such as dysphagia, were not statistically significantly associated with odds of cancer and were excluded from our model. Recent studies showed that dysphagia could be, more often, considered as a GERD symptom, rather than esophageal cancer. [62] Owing the fact that the majority (72.6%) of study participants were GERD patients, finding no significant associations shouldn't be surprising. Furthermore, most esophageal cancer patients with dyspepsia are relatively elderly patients and dysphagia indicate that the cancer is beyond the point of curability, [63] therefore, use of this risk-prediction model would perhaps not be a significant risk to them.

Making a decision as to perform endoscopy versus provide other treatments first requires a careful cost-benefit analysis. Such analysis depends partly on the risk-prediction model but it needs to take into consideration other factors such as probability of missing a potentially curable cancer if the treatment is delayed by a few weeks; additional benefits of endoscopy such as diagnosis of conditions other than cancer, as well as its harms and cost; prevalence of cancer and other underlying diseases causing dyspepsia; availability of endoscopic facilities; and its cost in any specific health setting.

In the United States and most European countries where *H. pylori* and UGI malignancy prevalence is low, while the cost of upper GI endoscopy is very high, cost-effectiveness analysis usually reveal that initial endoscopy is not beneficial and a test and treat approach is the most cost-efficient strategy. [64] However, applying a validated risk-prediction model to find high-risk patients for UGI malignancies and targeting them for performing endoscopy might be an alternative strategy to better compare the cost-benefit of two approaches. Furthermore, for Asian countries such as China and Iran, this recommendation, probably would not be applicable. [61] Unless, non-invasive and cheaper tests become available in countries like Iran, where endoscopy is widely available with a relatively low cost, prompt endoscopy may be recommended in all dyspeptic patients older than 50, with weight loss, or with any additional alarm symptoms.

The strengths of our study are relatively large sample size, availability of data on at least 10 predictors, and constructing risk-prediction model. A limitation of the study is that the risk-prediction model was based on a development (training) set and there was no external validation set.

In summary, none of the predictors that we studied demonstrated high diagnostic accuracy. Using age, alarm symptoms, family history of UGI cancer and smoking, we were able to construct a useful risk-prediction model that distinguished between high-risk and low-risk individuals with a ROC curve AUC of 0.85 and adequate overall calibration and model fit measures. However, the decision on how to use this model will depend on cost-benefit analytic models that depend on several other factors.

## Supporting Information

Table S1 **Diagnostic accuracy measures of alarm symptoms on UGI malignancies.**
(DOC)

Table S2 **Diagnostic accuracy measures for having only one, two, more than two and at least one alarm symptom in all ages, as well as according to age categories.**
(DOC)
