# Supplementary material for: Diagnostic Accuracy of Age and Alarm Symptoms for Upper GI Malignancy in Patients with Dyspepsia in a GI Clinic: A 7-Year Cross-Sectional Study
Source: PLoS One. 2012 Jun 13;7(6):e39173. doi: 10.1371/journal.pone.0039173 (PMC3374763; doi:10.1371/journal.pone.0039173)
Supplement: Table S1 — Diagnostic accuracy measures of alarm symptoms on UGI malignancies. (DOC) [file pone.0039173.s001.doc]

Table S1 Diagnostic accuracy measures of alarm symptoms on UGI malignancies

| Alarm symptoms | | | Sensitivity% (95%CI) | Specificity% (95%CI) | PPV%  (95%CI) | NPV%  (95%CI) | PDLR  (95%CI) | NDLR  (95%CI) | Diagnostic OR  (95%CI) |
| --- | --- | --- | --- | --- | --- | --- | --- | --- | --- |
| **Weight loss** | | | | | | | | | |
|  | **All ages** | | **43.7 (33.1 - 54.7)** | **90.0 (88.8 - 91.1)** | **12.1 (8.71 - 16.2)** | **98.1 (97.5 - 98.6)** | **4.37 (3.36 - 5.68)** | **0.63 (0.52 - 0.75)** | **6.98 (4.50 - 10.8)** |
|  | **In age groups** | |  |  |  |  |  |  |  |
|  |  | ≤35 yrs old | 57.1 (18.4 - 90.1) | 87.5 (85.2 - 89.5) | 3.15 (0.86 - 7.87) | 99.7 (99.0 - 99.9) | 4.56 (2.35 - 8.85) | 0.49 (0.21 - 1.15) | 9.31 (2.30 - 37.6) |
|  |  | 36-49 yrs old | 22.2 (6.41 - 47.6) | 92.2 (90.3 - 93.8) | 5.19 (1.43 - 12.8) | 98.4 (97.3 - 99.1) | 2.85 (1.17 - 6.95) | 0.84 (0.66 - 1.08) | 3.38 (1.14 - 10.0) |
|  |  | 50-64 yrs old | 34.8 (16.4 - 57.3) | 91.8 (89.3 - 93.8) | 13.8 (6.15 - 25.4) | 97.4 (95.7 - 98.5) | 4.23 (2.28 - 7.86) | 0.71 (0.53 - 0.96) | 5.95 (2.46 - 14.4) |
|  |  | ≥65 yrs old | 56.8 (39.5 - 72.9) | 86.5 (81.3 - 90.7) | 41.2 (27.6 - 55.8) | 92.3 (87.8 - 95.5) | 4.20 (2.72 - 6.49) | 0.50 (0.34 - 0.73) | 8.40 (3.98 - 17.8) |
| **GI bleeding** | | | | | | | | | |
|  | **All ages** | | **24.1 (15.6 - 34.5)** | **85.8 (84.5 - 87.1)** | **5.10 (3.18 - 7.69)** | **97.3 (96.6 - 97.9)** | **1.70 (1.16 - 2.50)** | **0.88 (0.78 - 1.00)** | **1.93 (1.17 - 3.17)** |
|  | **In age groups** | |  |  |  |  |  |  |  |
|  |  | ≤35 yrs old | 28.6 (3.67 - 71.0) | 82.8 (80.3 - 85.1) | 1.17 (0.14 - 4.16) | 99.4 (98.6 - 99.8) | 1.66 (0.51 - 5.40) | 0.86 (0.54 - 1.38) | 1.92 (0.00 - 8.68) |
|  |  | 36-49 yrs old | 22.2 (6.41 - 47.6) | 86.6 (84.3 - 88.8) | 3.10 (0.85 - 7.75) | 98.3 (97.2 - 99.1) | 1.66 (0.69 - 4.01) | 0.90 (0.70 - 1.15) | 1.85 (0.63 - 5.45) |
|  |  | 50-64 yrs old | 30.4 (13.2 - 52.9) | 88.3 (85.5 - 90.8) | 8.97 (3.68 - 17.6) | 97.1 (95.3 - 98.3) | 2.61 (1.35 - 5.02) | 0.79 (0.60 - 1.03) | 3.31 (1.35 - 8.12) |
|  |  | ≥65 yrs old | 21.6 (9.83 - 38.2) | 88.3 (83.3 - 92.2) | 23.5 (10.7 - 41.2) | 87.1 (82.0 - 91.2) | 1.85 (0.91 - 3.76) | 0.89 (0.74 - 1.06) | 2.08 (0.88 - 4.95) |
| **Dysphagia** | | | | | | | | | |
|  | **All ages** | | **29.9 (20.5 - 40.6)** | **81.1 (79.6 - 82.6)** | **4.75 (3.13 - 6.89)** | **97.3 (96.6 - 98.0)** | **1.58 (1.14 - 2.20)** | **0.90 (0.75 - 0.99)** | **1.83 (1.15 - 2.92)** |
|  | **In age groups** | |  |  |  |  |  |  |  |
|  |  | ≤35 yrs old | 42.9 (9.90 - 81.6) | 81.0 (78.4 - 83.4) | 1.58 (0.33 - 4.54) | 99.5 (98.7 - 99.9) | 2.25 (0.95 - 5.35) | 0.71 (0.37 - 1.34) | 3.19 (0.79 - 12.8) |
|  |  | 36-49 yrs old | 22.2 (6.41 - 47.6) | 81.3 (78.7 - 83.8) | 2.23 (0.61 - 5.62) | 98.2 (97.0 - 99.0) | 1.19 (0.50 - 2.85) | 0.96 (0.75 - 1.23) | 1.24 (0.42 - 3.64) |
|  |  | 50-64 yrs old | 21.7 (7.46 - 43.7) | 80.1 (76.7 - 83.2) | 3.97 (1.30 - 9.02) | 96.4 (94.4 - 97.9) | 1.09 (0.49 - 2.41) | 1.00 (0.78 - 1.22) | 1.12 (0.42 - 2.97) |
|  |  | ≥65 yrs old | 32.4 (18.0 - 49.8) | 83.8 (78.3 - 88.4) | 25.0 (13.6 - 39.6) | 88.2 (83.0 - 92.2) | 2.00 (1.15 - 3.48) | 0.81 (0.64 - 1.02) | 2.48 (1.16 - 5.33) |
| **Recurrent vomiting** | | | | | | | | | |
|  | **All ages** | | **27.6 (18.5 - 38.2)** | **89.2 (88.0 - 90.3)** | **7.45 (4.83 - 10.9)** | **97.5 (96.8 - 98.1)** | **2.55 (1.79 - 3.65)** | **0.81 (0.71 - 0.92)** | **3.15 (1.95 - 5.09)** |
|  | **In age groups** | |  |  |  |  |  |  |  |
|  |  | ≤35 yrs old | 28.6 (3.67 - 71.0) | 83.7 (81.2 - 86.0) | 1.23 (0.15 - 4.39) | 99.4 (98.6 - 99.8) | 1.75 (0.54 - 5.71) | 0.85 (0.53 - 1.36) | 2.06 (0.00 - 9.28) |
|  |  | 36-49 yrs old | 27.8 (9.69 - 53.5) | 92.8 (91.0 - 94.4) | 6.94 (2.29 - 15.5) | 98.5 (97.5 - 99.2) | 3.88 (1.78 - 8.46) | 0.78 (0.58 - 1.04) | 4.99 (1.80 - 13.9) |
|  |  | 50-64 yrs old | 30.4 (13.2 - 52.9) | 92.8 (90.4 - 94.7) | 13.7 (5.70 - 26.3) | 97.2 (95.6 - 98.4) | 4.21 (2.13 - 8.30) | 0.75 (0.57 - 0.98) | 5.61 (2.25 - 14.0) |
|  |  | ≥65 yrs old | 27.0 (13.8 - 44.1) | 87.8 (82.8 - 91.8) | 27.0 (13.8 - 44.1) | 87.8 (82.8 - 91.8) | 2.22 (1.18 - 4.20) | 0.83 (0.68 - 1.02) | 2.67 (1.19 - 6.06) |

PPV: positive predictive value, NPV: negative predictive value, PDLR: positive diagnostic likelihood ratio, NDLR: negative diagnostic likelihood ratio
