# Supplementary material for: Diagnostic Accuracy of Age and Alarm Symptoms for Upper GI Malignancy in Patients with Dyspepsia in a GI Clinic: A 7-Year Cross-Sectional Study
Source: PLoS One. 2012 Jun 13;7(6):e39173. doi: 10.1371/journal.pone.0039173 (PMC3374763; doi:10.1371/journal.pone.0039173)
Supplement: Table S2 — Diagnostic accuracy measures for having only one, two, more than two and at least one alarm symptom in all ages, as well as according to age categories. (DOC) [file pone.0039173.s002.doc]

Table S1 Diagnostic accuracy measures for having only one, two, more than two and at least one alarm symptom in all ages, as well as according to age categories

| Alarm symptoms | | | Sensitivity% (95%CI) | Specificity% (95%CI) | PPV%  (95%CI) | NPV%  (95%CI) | PDLR  (95%CI) | NDLR  (95%CI) | Diagnostic OR  (95%CI) |
| --- | --- | --- | --- | --- | --- | --- | --- | --- | --- |
| **Only one AS** | | |  |  |  |  |  |  |  |
|  | **All ages** | | **42.0 (28.2 - 56.8)** | **69.1 (67.2 - 70.9)** | **2.71 (1.68 - 4.11)** | **98.3 (97.6 - 98.9)** | **1.36 (0.98 - 1.89)** | **0.84 (0.66 - 1.06)** | **1.62 (0.92 - 2.84)** |
|  | **In age groups** | |  |  |  |  |  |  |  |
|  |  | ≤35 yrs old | 66.7 (9.43 - 99.2) | 66.2 (62.9 - 69.4) | 0.70 (0.08 - 2.52) | 99.8 (99.0 - 100) | 1.97 (0.88 - 4.41) | 0.50 (0.10 - 2.50) | 3.91 (0.51 - ⇸⇸) |
|  |  | 36-49 yrs old | 33.3 (9.92 - 65.1) | 71.4 (68.2 - 74.4) | 1.63 (0.45 - 4.11) | 98.7 (97.4 - 99.4) | 1.16 (0.52 - 2.61) | 0.93 (0.62 - 1.40) | 1.25 (0.40 - 3.93) |
|  |  | 50-64 yrs old | 35.7 (12.8 - 64.9) | 70.7 (66.7 - 74.4) | 2.99 (1.00 - 6.85) | 97.7 (95.8 - 99.0) | 1.22 (0.60 - 2.49) | 0.91 (0.61 - 1.35) | 1.34 (0.46 - 3.87) |
|  |  | ≥65 yrs old | 45.0 (23.1 - 68.5) | 66.7 (59.6 - 73.2) | 12.0 (5.64 - 21.6) | 92.3 (86.7 - 96.1) | 1.35 (0.80 - 2.28) | 0.82 (0.55 - 1.24) | 1.64 (0.66 - 4.05) |
| **Only two ASs** | | |  |  |  |  |  |  |  |
|  | **All ages** | | **46.3 (32.6 - 60.4)** | **87.6 (86.1 - 89.1)** | **9.51 (6.25 - 13.7)** | **98.3 (97.6 - 98.9)** | **3.74 (2.74 - 5.11)** | **0.61 (0.48 - 0.78)** | **6.11 (3.54 - 10.6)** |
|  | **In age groups** | |  |  |  |  |  |  |  |
|  |  | ≤35 yrs old | 75.0 (19.4 - 99.4) | 84.9 (81.9 - 87.6) | 2.97 (0.62 - 8.44) | 99.8 (99.0 - 100) | 4.97 (2.75 - 9.01) | 0.29 (0.05 - 1.61) | 16.9 (2.39 - ⇸⇸) |
|  |  | 36-49 yrs old | 38.5 (13.9 - 68.4) | 88.8 (86.2 - 91.1) | 6.17 (2.03 - 13.8) | 98.7 (97.4 - 99.4) | 3.44 (1.67 - 7.06) | 0.69 (0.45 - 1.07) | 4.96 (1.66 - 14.8) |
|  |  | 50-64 yrs old | 40.0 (16.3 - 67.7) | 89.7 (86.4 - 92.4) | 11.8 (4.44 - 23.9) | 97.7 (95.8 - 99.0) | 3.87 (1.96 - 7.62) | 0.67 (0.44 - 1.01) | 5.78 (2.05 - 16.4) |
|  |  | ≥65 yrs old | 47.6 (25.7 - 70.2) | 87.4 (81.0 - 92.3) | 34.5 (17.9 - 54.3) | 92.3 (86.7 - 96.1) | 3.78 (2.05 - 7.00) | 0.60 (0.40 - 0.90) | 6.32 (2.41 - 16.6) |
| **More than two ASs** | | |  |  |  |  |  |  |  |
|  | **All ages** | | **29.3 (16.1 - 45.5)** | **95.4 (94.3 - 96.3)** | **12.9 (6.85 - 21.5)** | **98.3 (97.6 - 98.9)** | **6.39 (3.79 - 10.8)** | **0.74 (0.61 - 0.90)** | **8.62 (4.29 - 17.3)** |
|  | **In age groups** | |  |  |  |  |  |  |  |
|  |  | ≤35 yrs old | 50.0 (1.26 - 98.7) | 91.7 (89.2 - 93.8) | 1.96 (0.05 - 10.4) | 99.8 (99.0 - 100) | 6.02 (1.47 - 24.7) | 0.54 (0.14 - 2.18) | 11.0 (0.00 - ⇸⇸) |
|  |  | 36-49 yrs old | 11.1 (0.28 - 48.2) | 97.6 (96.0 - 98.6) | 6.25 (0.16 - 30.2) | 98.7 (97.4 - 99.4) | 4.58 (0.67 - 31.0) | 0.91 (0.72 - 1.15) | 5.03 (0.00 - 33.6) |
|  |  | 50-64 yrs old | 25.0 (5.49 - 57.2) | 97.3 (95.1 - 98.6) | 21.4 (4.66 - 50.8) | 97.7 (95.8 - 99.0) | 9.11 (2.91 - 28.5) | 0.77 (0.56 - 1.07) | 11.8 (3.07 - 46.7) |
|  |  | ≥65 yrs old | 38.9 (17.3 - 64.3) | 96.4 (91.7 - 98.8) | 58.3 (27.7 - 84.8) | 92.3 (86.7 - 96.1) | 10.7 (3.78 - 30.1) | 0.63 (0.44 - 0.92) | 16.8 (4.78 - 59.2) |
| **At least one AS** | | |  |  |  |  |  |  |  |
|  | **All ages** | | **66.7 (55.7 - 76.4)** | **61.1 (59.3 - 62.9)** | **5.13 (3.92 - 6.58)** | **98.3 (97.6 - 98.9)** | **1.71 (1.47 - 2.00)** | **0.54 (0.40 - 0.73)** | **3.14 (2.01 - 4.93)** |
|  | **In age groups** | |  |  |  |  |  |  |  |
|  |  | ≤35 yrs old | 85.7 (42.1 - 99.6) | 56.2 (53.0 - 59.3) | 1.38 (0.51 - 2.97) | 99.8 (99.0 - 100) | 1.96 (1.43 - 2.67) | 0.25 (0.04 - 1.56) | 7.70 (1.21 - ⇸⇸) |
|  |  | 36-49 yrs old | 55.6 (30.8 - 78.5) | 64.4 (61.3 - 67.5) | 2.92 (1.41 - 5.30) | 98.7 (97.4 - 99.4) | 1.56 (1.02 - 2.38) | 0.69 (0.41 - 1.16) | 2.26 (0.91 - 5.62) |
|  |  | 50-64 yrs old | 60.9 (38.5 - 80.3) | 64.1 (60.2 - 68.0) | 6.03 (3.34 - 9.92) | 97.7 (95.8 - 99.0) | 1.70 (1.20 - 2.40) | 0.61 (0.36 - 1.02) | 2.78 (1.21 - 6.39) |
|  |  | ≥65 yrs old | 70.3 (53.0 - 84.1) | 59.5 (52.7 - 66.0) | 22.4 (15.2 - 31.1) | 92.3 (86.7 - 96.1) | 1.73 (1.33 - 2.26) | 0.50 (0.30 - 0.83) | 3.47 (1.65 - 7.27) |

PPV: positive predictive value, NPV: negative predictive value, PDLR: positive diagnostic likelihood ratio, NDLR: negative diagnostic likelihood ratio
